# Prevalence of overweight, obesity, and associated factors among healthcare workers in the Gaza Strip, Palestine: A cross-sectional study

**DOI:** 10.3389/fpubh.2023.1129797

**Published:** 2023-02-23

**Authors:** Joma Younis, Hong Jiang, Yahui Fan, Lina Wang, Zhaofang Li, Majed Jebril, Mei Ma, Le Ma, Mao Ma, Zhaozhao Hui

**Affiliations:** ^1^School of Public Health, Xi'an Jiaotong University Health Science Center, Xi'an, China; ^2^Al-Rantisi Pediatric Specialized Hospital, Ministry of Health, Gaza, Palestine; ^3^The First Affiliated Hospital, Xi'an Jiaotong University Health Science Center, Xi'an, China

**Keywords:** overweight, obesity, healthcare workers, prevalence, Gaza Strip-Palestine

## Abstract

**Background:**

Overweight and obesity are multifactorial conditions that are prevalent in developing and developed countries. They are emerging as a significant public health concern among healthcare workers (HCWs). We aimed to estimate the prevalence of overweight and obesity and their associated factors among HCWs in the Gaza Strip.

**Methods:**

A cross-sectional study was conducted to recruit 1,850 HCWs aged 22 years and older. Interviews were carried out to collect sociodemographic information, nutritional information, and physical activity. Anthropometric measurements [height, weight, and waist circumference] were conducted with the HCWs. The body mass index was computed to determine the prevalence of overweight and obesity. Chi-square, *t*-test, and one-way ANOVA were used to compare the variables, and logistic regression was used to examine the associated factors of overweight and obesity.

**Results:**

The combined prevalence of overweight and obesity among HCWs was 65%. The result of logistic regression showed the risk of being overweight and obesity increased within the age group of 40–49 years (OR = 3.20; 95% CI: 2.37–4.32; *P* < 0.001). Male participants had more risk of obesity than female participants (OR = 1.77; 95% CI: 1.45–2.15). Married participants had a significantly higher risk of being overweight and obese (OR = 2.52; 95% CI: 2.05–3.28; *P* = 0.001). Increased monthly income was significantly associated with the risk of being overweight and obese (OR = 2.16; 95% CI: 1.22–3.83; *P* = 0.008). In addition, hypertension (OR = 2.49; 95% CI: 1.65–3.78; *P* < 0.001) and type 2 diabetes (OR = 2.42; 95% CI: 1.21–4.85; *P*= 0.012) were associated with overweight and obesity. Finally, a family history of NCDs was associated with overweight and obesity (OR = 1.69; 95% CI: 1.38–2.07; *P* < 0.001).

**Conclusion:**

This study showed a high prevalence of overweight and obesity among HCWs. Age, monthly income, marital status, known hypertension, type 2 diabetes, and eating habits were associated with the prevalence of overweight and obesity compared to other variables that were not associated with overweight and obesity such as profession, vegetables, fruit consumption, and physical activity. Urgent action is needed to tackle overweight and obesity among HCWs.

## Introduction

Around the world, 650 million adults, 340 million adolescents, and 39 million children are obese. This estimation is still rising. According to the WHO, 167 million adults and children will experience deteriorating health by 2025 as a result of being overweight or obese (1). Obesity is a significant contributor to poor health, including type 2 diabetes, cardiovascular disease, cancer, decreased life expectancy, and mortality (2), which could impact all classes of communities.

Most body systems, including the endocrine, gastrointestinal, neurological, and cardiovascular systems, are severely impacted by overweight and obesity, increasing a person's risk of contracting infectious diseases (3). Obesity-related complications increase the number of morbidities that need to be managed by the declining number of healthcare professionals (4). Healthcare workers (HCWs) who have direct contact with patients and often influence their behaviors ought to have a healthy physique to show a good role model in front of patients, and this revealed the importance of a healthy physique toward disease prevention (5). Overweight and obese HCWs might have difficulty counseling the patients even if the patients clinically state with weight increase (6), which could be one of the barriers that can affect the patient consultation due to the same health issue.

Obesity and overweight rates are rapidly rising in developed and developing countries, including Palestine (7). Overweight [body mass index (BMI) of 25.0–29.9 kg/m^2^] and obesity (BMI ≥ 30 kg/m^2^) increases the risk of non-communicable diseases (NCDs) (8). A meta-analysis study in Middle East countries found that the prevalence of obesity and overweight was 21.17 and 33.14%, respectively (9). A recent survey conducted in Palestine concluded that the prevalence of overweight and obesity is 23.6 and 19.5% in the Gaza Strip and 26.1% in the West Bank (10).

Due to the Israeli siege, people in Gaza live in unfavorable and dangerous conditions. The political events there, where Palestinian refugees, in particular, and Palestinian lives, in general, are concerned, have contributed to widespread unemployment, malnutrition, food insecurity, a lack of income, and a continuous decline in the quality of care for all patient categories (11, 12). These factors undoubtedly could have an impact on people with NCDs. Healthcare workers are exposed to a wide range of difficulties that prevent the provision of health services as required, including lack of human resources, lack of medical supplies, workload, and financial burden, and these factors may negatively affect the health of health sector workers and lead to the development of work-related illness (13).

Understanding the viewpoints of healthcare professionals is crucial before creating a successful healthcare intervention (14). A healthy society depends on the efforts of an important set of professionals, the HCWs (15). Unfortunately, little health research has been done on managing NCDs. Previous studies revealed that sedentary jobs, long periods of sitting, and shift work all greatly raise the risk of obesity (16). Healthcare providers include certified medical personnel (e.g., doctors, nurses, medical scientists, pharmacists, and technicians) and non-clinical support staff (e.g., the administrative class) (17). Because of their specialized training, healthcare professionals are supposed to have a high level of knowledge and awareness of their health condition and the effects of lifestyle changes on their health (18, 19). In addition, HCWs are responsible for promoting appropriate lifestyle changes that affect disease prevention and serve as role models for the general population by leading healthy lifestyles (20).

Therefore, few studies have been conducted to evaluate overweight, obesity, and the risk factors linked to these conditions among Palestinian HCWs working in the Gaza Strip. Because they are overlooked in research studies while being identified as high-risk populations, this study was done to evaluate the prevalence of overweight and obesity and its associated factors among HCWs.

## Material and methods

### Study design and setting

A cross-sectional study was conducted from Feb to May 2020 in the Gaza Strip, Palestine, among a representative sample of Palestinian HCWs (physicians, nurses, paramedics, and non-medical) who work in hospitals and the primary healthcare (PHCs) of the ministry of health. Gaza Strip is divided into five smaller governorates, which include North Gaza, Gaza City, Mid Zone, Khan Younis, and Rafah. The whole number of hospitals was 10 and PHCs was 51 (21). The study sample was distributed according to the number of hospitals and PHCs in each governorate.

### Sampling methods and participants

The consistency and completeness of all 1,900 responses were rigorously checked. The final analysis had 1,850 responses after 50 were excluded since they were considered incomplete or inconsistent. The participants were selected by multistage stratified random sampling. First, we dived the population into five governorates, and second, we selected two hospitals and three PHCs for each governorate. Considering the population distribution in each area, we have increased the sample size to be representative of the overall HCWs in the Gaza Strip. The sample size was determined using the formula *n* = Z^2^ P (1–P)/d^2^ at 95% CI (22).

### Study criteria

Our inclusion criteria were: All HCWs working at the ministry of health in the Gaza Strip, with at least 1 year of experience, aged 22 years and older. Pregnant and lactating women, in addition to those workers under an unemployment program, were excluded.

### Data collection

Using a self-constructed face-to-face interview questionnaire, data were collected with multistage stratified random sampling. The data included detailed demographic and socioeconomic information (sex, marital status, educational levels, workplace, experience, monthly income, etc.), lifestyle involved (sleep duration, workload, work routine, and physical activity [measured by the International Physical Activity Questionnaire, IPAQ-short version]) (23); the health profile involved [dietary patterns adapted from questions used in the food frequency questionnaire (24, 25)], menstrual cycle, family history of the disease, medical records, etc. After analyzing the literature on the subject, the questionnaire was well-prepared. The questionnaire's validity was tested by sending the completed questionnaire and a cover letter explaining the study's goal to 10 experts in various health professions (associated professors, hospital directors, managers of health departments, and academic teachers) who were asked to comment on the questionnaire. From the original English edition, all questions were translated into Arabic (forward and English and Arabic mother-tongue speakers performed backward translations). The study's research team consisted of five workers, physicians, and nurses, who collected the questionnaires by filling out the printed sheets.

Expert nurses measured each participant's anthropometric data by using standard protocols (26, 27), and height and weight were measured using stadiometers and weighing scales, respectively (26). A measuring tape was positioned 1 cm below the umbilicus and at the iliac crest to measure the circumferences of the waist and hips, respectively (27). The body mass index (BMI) formula (kg/m^2^) = Weight (kg)/Height squared (m^2^) (28) was used to calculate the BMI. We defined obesity according to WHO criteria; where underweight people had a BMI of <18.5 kg/m^2^, normal weight had a BMI of 18.5–24.9 kg/m^2^, overweight had a BMI of 25.0–29.9 kg/m^2^, and obese were over 30.0 kg/m^2^ (29).

The blood pressure (BP) values were taken with a sphygmomanometer. After participants had rested in a sitting position for at least 10 min, experienced nurses took two measurements on the right arm at a properly sized cuffed 1-min interval, with the arm supported at heart level and feet flat on the floor (30).

### Ethical considerations

All subjects signed consent forms before participating in the study. The ethical committee of Xi'an Jiao tong University Health Science Center approved the study, which was carried out following the Declaration of Helsinki. The Palestinian Health Research Committee at the Directorate General of Human Resources Development, Ministry of Health, Gaza (PHRC/HC/663/19) approved the protocol. The data were analyzed in an anonymous and non-linked manner, with no participant names being used. Moreover, there are no physical risks as there is no intervention such as blood sampling during the study.

### Statistical analysis

SPSS V.26 (Statistical package of social science) was used to carry out all data analyses. Continuous variables were represented by mean values and standard deviations (SD), while categorical variables were described by frequency and percentage. For categorical and continuous variables, chi-square and *t*-tests were used. If three or more groups were studied, one-way variance analysis (ANOVA) was used to compare demographic characteristics between groups.

The odds ratio (OR) and 95% confidence interval (CI) of overweight and obesity were calculated by using univariate logistic regression, with the predictors associated (gender, age, marital status, work experience, monthly income, known hypertension, type 2 diabetes, and family history of NCDs). The statistical significance was set as a two-sided level, *P* < 0.05.

## Results

### Sociodemographic characteristics of the HCWs

A total of 1,850 HCWs were included in this study, 1,146 (61.9%) were male participants, 704 (38.1%) were female participants, and most of the participants were in the age group (30–39) with 49.3%. About 78.1% of participants were married, while 68.6% had a first degree (Bachelor's). The HCWs included 226 physicians (12.2%), 1,208 nurses (65.3%), 334 paramedics (18.1%), and 82 non-medical (4.4%). Most of the participants (33.8%) had work experience of 10–15 years. The average monthly income of most participants was less than (2,000 NIS) per month at 61.8%.

The means anthropometric measurements for the participants were as follow: height 170.39 ± (8.86) cm, weight 78.63 ± (14.92) Kg, BMI 27.09 ± (4.77) Kg/m^2^, waist circumference 107.14 ± (11.63) cm, hip circumference 99.83 ± (7.02) cm, systolic blood pressure (SBP) 118.16 ± (10.13) mmHg, and diastolic blood pressure (DBP) 73.04 ± (10.01) mmHg. Moreover, 65% of the participants were overweight or obese. BMI had significant associations with gender, age, marital status, profession, experience, monthly income, and anthropometric measurements (weight, height, waist circumference, hip circumference, SBP, and DBP) (*P* < 0.05; Table 1).

**Table 1:** Baseline characteristics of HCWs in the Gaza Strip by BMI category (*N* = 1,850).

**Variable**	**All**	**Underweight**	**Normal**	**Overweight**	**Obese**	***P*-value**
	***n* =1,850**	***n* = 18 (1.0%)**	***n* = 630 (34.0%)**	***n* = 727 (39.3%)**	***n* = 475 (25.7%)**	
**Gender**						
Male	1,146	8 (0.7)	336 (29.3)	490 (42.8)	312 (27.2)	<0.001^a^
Female	704	10 (1.4)	294 (41.7)	237 (33.7)	163 (23.2)	
**Age group (years)**						
22–29	334	2 (0.6)	184 (55.1)	98 (29.3)	50 (15.0)	<0.001^a^
30–39	912	10 (1.1)	304 (33.3)	390 (42.8)	208 (22.8)	
40–49	440	6 (1.4)	118 (26.8)	184 (41.8)	132 (30.0)	
50–61	164	-	24 (14.6)	55 (33.6)	85 (51.8)	
**Marital status**						
Unmarried	406	4 (1.0)	201 (49.5)	135 (33.2)	66 (16.3)	<0.001^a^
Married	1,444	14 (1.0)	429 (29.7)	592 (41.0)	409 (28.3)	
**Educational level**						
Diploma	354	5 (1.4)	129 (36.5)	142 (40.1)	78 (22.0)	0.258^a^
Bachelor	1,269	12 (0.9)	433 (34.1)	498 (39.3)	326 (25.7)	
Post-graduated	227	1 (0.4)	68 (30.0)	87 (38.3)	71 (31.3)	
**Profession**						
Physician	226	4 (1.8)	68 (30.1)	94 (41.6)	60 (26.5)	0.033^a^
Nurse	1,208	6 (0.5)	414 (34.3)	483 (40.0)	305 (25.2)	
Paramedics	334	8 (2.4)	112 (33.5)	122 (36.5)	92 (27.5)	
Non-medical	82	–	36 (43.9)	28 (34.1)	18 (22.0)	
**Experience (years)**						
<5	381	4 (1.0)	192 (50.4)	132 (34.7)	53 (13.9)	<0.001^a^
5–10	575	6 (1.0)	192 (33.4)	238 (41.4)	139 (24.2)	
10–15	626	6 (1.0)	190 (30.3)	258 (41.2)	172 (27.5)	
>15	268	2 (0.8)	56 (20.9)	99 (36.9)	111 (41.4)	
**Monthly income (NIS)**						
<2,000	1,144	14 (1.2)	432 (37.8)	453 (39.6)	245 (21.4)	<0.001^a^
>2,000	706	4 (0.6)	198 (28.0)	274 (38.8)	230 (32.6)	
	**Mean (SD)**	**Mean (SD)**	**Mean (SD)**	**Mean (SD)**	**Mean (SD)**	
Height	170.39 (8.86)	170.44 (10.39)	170.05 (8.97)	171.46 (8.95)	169.19 (8.33)	<0.001^b^
Weight	78.63 (14.92)	52.67 (6.41)	65.36 (8.45)	80.28 (8.61)	94.69 (11.68)	<0.001^b^
BMI	27.09 (4.77)	18.07 (0.37)	22.53 (1.64)	27.24 (1.24)	33.25 (3.78)	<0.001^b^
WC	107.14 (11.63)	82.22 (2.41)	97.25 (5.58)	110.51 (9.96)	116.03 (9.20)	<0.001^b^
HC	99.83 (7.02)	82.72 (4.53)	95.61 (5.08)	100.32 (5.29)	105.30 (7.02)	<0.001^b^
SBP	118.16 (10.13)	113.33 (8.40)	115.72 (8.76)	118.51 (10.02)	121.04 (11.18)	<0.001^b^
DBP	73.04 (10.01)	67.78 (9.42)	70.40 (9.44)	73.55 (9.48)	75.94 (1.61)	<0.001^b^

BMI, Body mass index; DBP, Diastolic blood pressure; SBP, Systolic blood pressure. ^*a*^ Chi-square test; ^*b*^ ANOVA. The P-value was considered statistically significant when P < 0.05.

### Prevalence of overweight and obesity among HCWs

Based on gender, the prevalence of overweight and obesity among HCWs was 43.4% for male participants and 21.6% for female participants. Moreover, the total prevalence in both genders was 65% for the whole study participants and 35% had normal weight. There is a statistical association between gender and BMI (*P* < 0.05; Table 1).

The prevalence of overweight and obesity among HCWs stratified by age group also offers the most age group at risk of overweight and obesity between 30 and 39 years with 32.32%, followed by 40 and 49 years with 17.09%, and the lowest prevalence for the age group of 50–61 years with 7.56%. There is a statistically significant association between age and BMI (*P* < 0.05; Table 1).

### Lifestyle factors of the healthcare workers and BMI

Approximately 18.9% of the study participants were smokers, and 65.5% had low physical activity. The consumption of fruits <3 days/week was 92.9%, and the consumption among overweight and obese, as well as for vegetables, was 96.6%, <3 days/week. However, there is no relationship between fruit/vegetable consumption and the prevalence of overweight, and obesity. While 91.6% of the participants ate three meals daily, 8.9% ate more than three times per week out of home, and 65.2% ate with more than five people. The prevalence of hypertension was 8.4%, type 2 diabetes was 2.9%, and family history of NCDs among participants was 67.6%. There are statistically significant associations between BMI and the number of meals, the number of meals eaten outdoors, the total number of members who ate together, known hypertension, type 2 diabetes, and family history of NCDs (*P* < 0.05; Table 2).

**Table 2:** Lifestyle factors and medical history of HCWs in the Gaza Strip by BMI category (*n* = 1,850).

**Variable**	**All**	**Underweight**	**Normal**	**Overweight**	**Obese**	***P*-value**
	***n* = 1,850**	***N* = 18 (1.0%)**	***n* = 630 (34.0%)**	***n* = 727 (39.3%)**	***n* = 475 (25.7%)**	
**Smoking**						
Yes	350	2 (0.6)	100 (28.6)	152 (43.4)	96 (27.4)	
No	1,500	16 (1.1)	530 (35.3)	575 (38.3)	379 (25.3)	
**Physical activity**						
Low	1,211	12 (1.0)	421 (34.8)	456 (37.6)	322 (26.6)	0.353
Moderate	540	5 (0.9)	174 (32.2)	236 (43.7)	125 (23.2)	
Hight	99	1 (1.0)	35 (35.4)	35 (35.4)	28 (28.2)	
**Fruits consumption in a week**						
≤3 days	1,718	18 (1.0)	584 (34.0)	677 (39.4)	439 (25.6)	0.654
>3 days	132	–	46 (34.8)	50 (37.9)	36 (27.3)	
**Vegetable consumption in a week**						
≤3 days	1,788	18 (1.0)	604 (33.8)	707 (39.5)	459 (25.7)	0.457
>3 days	62	–	26 (41.9)	20 (32.3)	16 (25.8)	
**Number of meals (day)**						
≤3	1,694	16 (0.9)	570 (33.7)	685 (40.4)	423 (25.0)	0.009
>3	156	2 (1.3)	60 (38.5)	42 (26.9)	52 (33.3)	
**How many days do you usually eat at home every week?**						
≤3	164	4 (2.5)	44 (26.8)	62 (37.8)	54 (32.9)	0.015
>3	1,686	14 (0.8)	586 (34.8)	665 (39.4)	421 (25.0)	
**How many people do you usually eat together?**						
≤5	1,206	12 (1.0)	428 (35.5)	498 (41.3)	268 (22.2)	<0.001
>5	644	6 (0.9)	202 (31.4)	229 (35.6)	207 (32.1)	
**Known hypertension**						
Yes	155	–	29 (18.7)	55 (35.5)	71 (45.8)	
No	1,695	18 (1.1)	601 (35.5)	672 (39.6)	404 (23.8)	
**Known type 2 diabetes**						
No	1,796	15 (0.8)	623 (34.7)	705 (39.3)	453 (25.2)	<0.001
Yes	54	3 (5.6)	7 (13.0)	22 (40.7)	22 (40.7)	
**Family history of NCDs**						
Yes	1,250	8 (0.6)	380 (30.4)	505 (40.4)	357 (28.6)	
No	600	10 (1.7)	250 (41.7)	222 (37.0)	118 (19.6)	

The P-value was considered statistically significant when P < 0.05. NCDs, Non-communicable diseases.

The prevalence of overweight and obesity among HCWs was 65%. Approximately 21.6% of them were female participants and 43.4% were male participants. The most age group of overweight and obesity was 30–39 years with 32.3%. Moreover, nurses were the most HCWs who had overweight and obese, with 42.6%. In addition, the prevalence of type 2 diabetes and hypertension was higher in a group of overweight and obese, thus, there is a statistically significant association between BMI and type 2 diabetes and hypertension (*P* < 0.05; Table 2).

### Association between overweight, obesity, and associated risk factors

Univariate analysis using logistic analysis showed the significant predictors associated (gender, age, marital status, work experience, monthly income, known hypertension, type 2 diabetes, and family history of NCDs) with overweight and obesity among HCWs.

The odds ratio of overweight and obesity increased 1.77 times for male participants than female participants (95% CI: 1.45–2.15). This relationship was statistically significant (*P* < 0.001). Moreover, the age group (40–49) was associated with overweight and obesity by almost three times (OR = 3.20; 95% CI: 2.37–4.32; *P* < 0.001). The OR of overweight and obesity among married participants was two times greater than that of unmarried participants (OR = 2.30; 95% CI: 1.84–2.88; *P* < 0.001).

Doctors were associated with an increase in overweight and obesity than non-medical professions by 1.67 times (OR = 1.67; 95% CI: 0.99–2.81). This relationship was not statistically significant (*P* = 0.051). The work experience increased overweight and obesity by 3.8 times for more than 15 years (OR = 3.83; 95% CI: 2.69–5.46; *P* < 0.001). Furthermore, the increase in monthly income was associated with overweight and obesity by 1.59 times (OR = 1.59; 95% CI: 1.30–1.95; *P*<*0.001*).

Hypertension was strongly associated with overweight and obesity (OR = 2.49; 95% CI: 1.65–3.78; *P* < 0.001) as well as overweight and obesity was associated with type 2 diabetes (OR = 2.42; 95% CI: 1.21–4.85; *P* = 0.012). Finally, a family history of NCDs was associated with overweight and obesity (OR = 1.69; 95% CI: 1.38–2.07; *P* < 0.001; Table 3).

**Table 3:** The association between HCWs' overweight and obesity and associated factors.

**Variables**	**Regression coefficient B**	**OR (95% CI)**	**Wald statistics (df)**	***P*-value**
**Gender**				
Male	0.572	1.77 (1.45–2.15)	32.90	<0.001
Female		1		
**Age group**				
22–29		1		
30–39	0.873	2.393 (1.85–3.09)	44.83	<0.001
40–49	1.164	3.203 (2.37–4.32)	57.99	<0.001
50–61	1.992	7.331 (4.51–11.89)	65.12	<0.001
**Marital status**				
Unmarried		1		
Married	0.835	2.305 (1.84–2.88)	53.17	<0.001
**Profession**				
Physician	0.515	1.674 (0.99–2.81)	3.80	0.051
Nurse	0.384	1.468 (0.93–2.30)	2.77	0.096
Paramedics	0.333	1.396 (0.85–2.27)	1.77	0.182
Non-medical		1		
**Experience (years)**				
<5		1		
5–10	0.702	2.017 (1.54–2.62)	27.04	<0.001
10–15	0.843	2.324 (1.78–3.02)	39.66	<0.001
>15	1.340	3.836 (2.69–5.46)	55.59	<0.001
**Monthly income (NIS)**				
<2,000		1		
>2,000	0.466	1.594 (1.303–1.951)	20.50	<0.001
**Number of meals (day)**				
≤3		1		
>3	−0.044	0.957 (0.83–1.09)	0.42	0.515
**How many people do you usually eat together?**				
≤5		1		
>5	0.186	1.204 (0.98–1.47)	3.23	0.072
**Known hypertension**				
No		1		
Yes	0.916	2.499 (1.65–3.78)	18.66	<0.001
**Type 2 diabetes**				
No		1		
Yes	0.885	2.424 (1.21–4.85)	6.26	0.012
**Family history of NCDs**				
NO		1		
YES	0.530	1.699 (1.38–2.07)	26.68	<0.001

The P-value was considered statistically significant when P < 0.05. NCDs, Non-communicable diseases; NIS, New Israel shekel.

## Discussion

Obesity among HCWs is an important issue as it impacts the morbidity of HCWs. In the present study, the overall prevalence of overweight and obesity among HCWs was 65% (39.3% overweight and 25.7% obesity). These findings are comparable with the meta-analysis in the Middle Eastern countries, which reported the prevalence of overweight and obesity were 33.14 and 21.17%, respectively (9). Moreover, a previous study conducted in the Gaza Strip said the prevalence of obesity was 19.5% (10). The current research has found a higher prevalence of overweight and obesity among HCWs than the prevalence of overweight and obesity reported in the previous study. When compared to the overall adult population, healthcare workers have a higher prevalence of obesity. This may be because they are more susceptible to the disease due to irregular and extended work hours, poor food, and workplace stress.

There is a significant association between the elevation of the anthropometric measure (Height, weight, WC, HC, SBP, and DBP) and being overweight or obese. It was also noted that the participant's elevation in anthropometric measurements had a significant positive association with being overweight and obese. The higher prevalence of overweight and obesity among HCWs might be influenced by sedentary behaviors, which might be contributed to the work environments and the socioeconomic status that may encourage the adaption of less physical activity and eating habits (31, 32). One of the contributing factors may be eating a poor diet while watching TV, especially sugary snacks. Other researchers have discovered a link between screen time and sugary, high-energy snacks (33). Due to the work environment, the HCW's low frequency of intake of meals, low intake of fruits and vegetables, and low physical activity increased overweight and obesity. Further research is needed to investigate dietary habits and socioeconomic status and their association with overweight and obesity among HCWs.

Furthermore, the prevalence of overweight and obesity increased with age. Similar results were reported by Low et al. (34), Addo et al. (35), Kishawi et al. (36), and Firouzbakht et al. (37), that the prevalence of overweight and obesity increased as age increased. This places a future load of illness on the medical workforce. In addition, the previous study found that the estimated peak increase in the prevalence of overweight and obesity as age increased was 40–50 years in developing countries while it was 50–60 years in developed countries (34), which is in line with the current study. The peak increase in the prevalence of obesity with age was 30–39 years. In the present study, the peak age is lower than in the previous study. Most of the studies confirmed the association between age increasing and susceptibility to non-communicable diseases and overweight and obesity as one of the risk factors of illness. Overweight and obesity are associated with increased age due to decreased physical activity, routine daily activities, comorbidities, and dietary habits. Therefore, the high ratio of youth and the predominance of the HCWs category found in this study represent the actual age and job category distribution among HCWs in the Gaza Strip.

In addition, the findings from this study show that married participants had a higher prevalence of overweight and obesity than single participants. The results support the findings reported by Dagen et al. (38) and Tzotzas et al. (39) that married adults had a higher prevalence of being overweight and obese than single adults and hypothesized that the increase in BMI among married couples is due to the increased social support, along with regularly eating dense food that increases the risk of being overweight or obese (39).

Our results revealed that the prevalence of overweight and obese increased among patients who had hypertension and type 2 diabetes. In addition, participants with a positive family history of NCDs had a higher prevalence of overweight and obesity than those without a family history of NCDs, in which similar findings have been found in previous studies (40–43).

Similar to the findings of our investigation, numerous studies have shown that nurses had a higher risk of obesity than workers in other occupations. In their academic careers in Scotland and England (44, 45), it is covered that the risk of obesity was lower for different HCW job categories than for nurses. Unfortunately, Hegde et al. in their studies in Tamil Nadu, India, found results that contradicted our study, suggesting that the burden of obesity was greater among physicians than nurses (46). All the studies mentioned earlier agree that different job categories were associated with obesity among HCWs. However, the aforementioned studies did not share comparable sociodemographic populations to that of the Gaza Strip. Most studies among HCWs did not specifically analyze different types of HCW occupations and obesity. Hazmi et al. (47) only mentioned the overall prevalence of obesity without further analysis according to job category, while Mustafa et al. (48) and Ramli (49) only divided the occupations of HCWs into professional vs. ancillary jobs. In contrast, our study is the first in the Gaza Strip to work with HCWs and determine the prevalence of overweight and obesity and classify HCW occupations into four main groups, doctors, nurses, paramedical, and non-medical categories, with the prevalence of overweight and obesity. Thus, we know nurses are at a greater risk of becoming obese than doctors and other job categories in the Gaza Strip.

Our findings reported that eating behavior was significantly associated with increased overweight and obesity, thus, the participants <3 meals per day had increased the prevalence of overweight and obesity in other groups (Table 2). Incomparable to previous studies, eating behavior was reported as one of the leading factors in the development of overweight and obesity with carried gender and age differences (50–53). In addition, no local studies reported an association between eating behavior and the prevalence of obesity.

In addition, physical inactivity is identified as a risk factor for overweight and obesity (54, 55). However, the current study revealed that all healthcare workers with low or moderate activity had no significant association between physical activity and overweight and obesity. A similar result was reported by Firouzbakht et al. (37) and El Kishawi et al. (36). These previous studies recognized the low physical activity due to the working shift of HCWs, limited availability of exercise facilities, and workload.

However, the current study showed significant predictors associated with an increase in the prevalence of overweight and obesity among HCWs. These associated factors included gender, age, marital status, monthly income, known hypertension, type 2 diabetes, family history of NCDs, and elevation of anthropometric measurements. On the other hand, the current study did not find any significant associations between educational level, smoking status, physical activity, and fruit and vegetable consumption.

## Strengths and limitations of the study

This study has several strengths. The first study estimated the prevalence of overweight and obesity among HCWs in the Gaza Strip. This study defined the associated factors with an increase in the prevalence of overweight and obesity; therefore, this study will be a baseline for subsequent studies among HCWs. There are some limitations in the study that need to be considered. First, cross-sectional data do not explore the causal pathways that underlie the reported association. Second, no availability of previous studies about the prevalence of overweight and obesity to interpret our results. Third, recall bias is also possible by using food frequency. Fourth, the majority of our participants were nurses compared with other professions.

## Conclusion

This study showed a high prevalence of overweight and obesity among HCWs. Age, monthly income, marital status, known hypertension, type 2 diabetes, and eating habits were associated with the prevalence of overweight and obesity. These findings appear to show an emerging problem in HCWs. A wellness program should be developed by decision-makers throughout mass-level educational awareness campaigns for HCWs to prevent and manage the modifiable risk factors that increase overweight or obesity.

## Data availability statement

The original contributions presented in the study are included in the article/supplementary material, further inquiries can be directed to the corresponding author.

## Ethics statement

The studies involving human participants were reviewed and approved by Palestinian Health Research Committee at the Directorate General of Human Resources Development, Ministry of Health, Gaza (PHRC/HC/663/19). The patients/participants provided their written informed consent to participate in this study.

## Author contributions

LM, JY, and MaM generated the idea for the study and formulated a research plan. JY wrote the original draft preparation and reviewed and edited. MJ, HJ, YF, LW, ZL, ZH, and MeM revised the manuscript and interpreted the data and editing. LM and MaM supervised the study. All authors acquired, analyzed, and interpreted the data. All authors contributed to the article and approved the submitted version.
